# Supplementary figures and images for: Repeated origins, widespread gene flow, and allelic interactions of target-site herbicide resistance mutations
Source: eLife. 2022 Jan 17;11:e70242. doi: 10.7554/eLife.70242 (PMC8798060; doi:10.7554/eLife.70242)

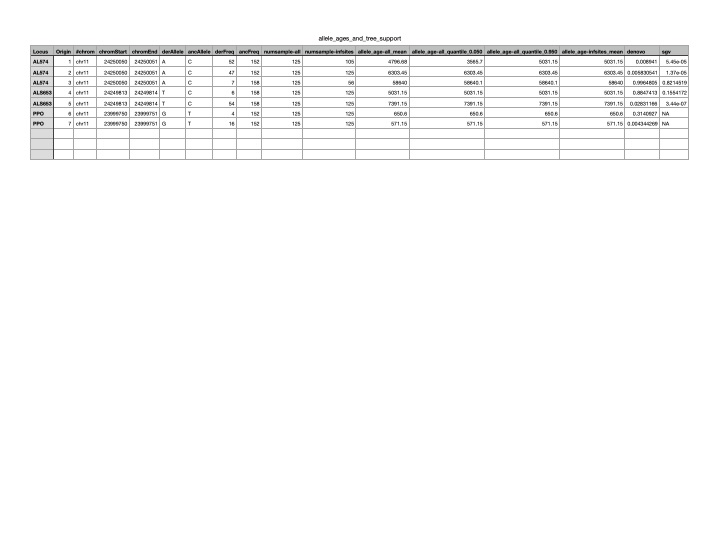

Supplement: Figure 3—source data 2. — Scaling was performed by dividing the raw age (based on an assumed Ne = 500,000) by 166.5894 (corresponding to the geometric mean contemporary Ne estimate of 83,294,700). [file elife-70242-fig3-data2.csv › preview.jpg]

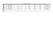

Supplement: Figure 3—source data 2. — Scaling was performed by dividing the raw age (based on an assumed Ne = 500,000) by 166.5894 (corresponding to the geometric mean contemporary Ne estimate of 83,294,700). [file elife-70242-fig3-data2.csv › preview-micro.jpg]

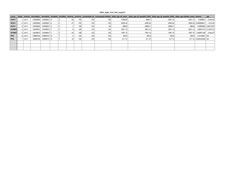

Supplement: Figure 3—source data 2. — Scaling was performed by dividing the raw age (based on an assumed Ne = 500,000) by 166.5894 (corresponding to the geometric mean contemporary Ne estimate of 83,294,700). [file elife-70242-fig3-data2.csv › preview-web.jpg]
